# Anomalous Spectral Features of a Neutral Bilayer Graphene

**DOI:** 10.1038/srep10025

**Published:** 2015-05-18

**Authors:** C.-M. Cheng, L.F. Xie, A. Pachoud, H.O. Moser, W. Chen, A.T.S. Wee, A.H. Castro Neto, K.-D. Tsuei, B. Özyilmaz

**Affiliations:** 1 National Synchrotron Radiation Research Center, 101 Hsin-Ann Road, Hsinchu, 30076, Taiwan; 2 Department of Physics, National University of Singapore, 2 Science Drive 3, 117542, Singapore; 3 NanoCore, 4 Engineering Drive 3, National University of Singapore 117576, Singapore; 4 Graduate School for Integrative Sciences and Engineering (NGS), National University of Singapore, 28 Medical Drive, 117456, Singapore; 5 Centre for Advanced 2D Materials and Graphene Research Centre, Faculty of Science, National University of Singapore, Block S14, Level 6, 6 Science Drive 2, 117546, Singapore; 6 Singapore Synchrotron Light Source, National University of Singapore, 5 Research Link 117603, Singapore; 7 Karlsruhe Institute of Technology (KIT), Network of Excellent Retired Scientists (NES) and Institute of Microstructure Technology (IMT), Postfach 3640, 76021 Karlsruhe, Germany; 8 Department of Physics, National Tsing Hua University, 101 Sec. 2, Kuang-Fu Road, Hsinchu 30013, Taiwan

## Abstract

Graphene and its bilayer are two-dimensional systems predicted to show exciting many-body effects near the neutrality point. The ideal tool to investigate spectrum reconstruction effects is angle-resolved photoemission spectroscopy (ARPES) as it probes directly the band structure with information about both energy and momentum. Here we reveal, by studying undoped exfoliated bilayer graphene with ARPES, two essential aspects of its many-body physics: the electron-phonon scattering rate has an anisotropic *k*-dependence and the type of electronic liquid is non-Fermi liquid. The latter behavior is evident from an observed electron-electron scattering rate that scales linearly with energy from 100 meV to 600 meV and that is associated with the proximity of bilayer graphene to a two-dimensional quantum critical point of competing orders.

The discovery of both single layer graphene (SLG) and bilayer graphene (BLG) has revolutionized the physics of low dimensional systems^1^,^2^,^3^ and led to new applications of nanoscale devices. The discovery of graphene helped create one of the most successful interdisciplinary research efforts driven by graphene’s outstanding electronic, chemical, optical, and mechanical properties. From a point of view of purely basic science, the massless, chiral, Dirac-like electronic spectrum of SLG with two linear energy bands touching each other at a single point (the Dirac point) has led to the observation of many exotic phenomena. BLG differs from SLG by only one additional layer, but adds an entirely new range of quantum phenomena based on the massive nature of its chiral Dirac fermions^4^. The spectrum of BLG comprises four massive Dirac bands (two conduction bands, two valence bands) that are the results of the broken sublattice symmetry generated by the rotation of sixty degrees of one layer with respect to the other (Bernal structure)^4^.

As in the case of SLG, the spectrum is gapless, but the bands are hyperbolic in accordance with a low energy Lorentz invariant theory (the dispersion relation is given by 
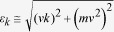
 where *v* is the Fermi-Dirac velocity, *k* is the 2D momentum, and *m* is the “rest” mass)^5^. In contrast to SLG, the absence of a gap in BLG is entirely due to an accidental degeneracy. A perpendicular electric field can thus serve to lift the degeneracy between the two layers and open up an energy gap^6^,^7^. The presence of a doping/field induced gap in BLG has been investigated in several experiments^6^,^7^,^8^,^9^,^10^. Measurements of ARPES showed directly the variation of the electronic structure of BLG^10^,^11^,^12^,^13^,^14^,^15^, but such experiments have focused mostly on epitaxial graphene grown on SiC. In graphene grown on the Si-face of SiC, charge transfer between the substrate and graphene shifts the Fermi level (E_F_) well above the Dirac energy (E_D_). Previous ARPES studies on quasi-free-standing graphene grown on the C-face of SiC^16^ and H-terminated SiC^17^ have been conducted to achieve electronically decoupled graphene layers, but focused on SLG. Little is hence known, at present, about the spectral properties of the unperturbed, neutral BLG. From a point of view of fundamental physics, highly doped BLG behaves almost as an ordinary metal and is described by Fermi liquid theory, but in undoped BLG, such as mechanically exfoliated BLG (ExBLG), this description is expected to break down; the system can be considered as quantum critical, when E_F_ resides at E_D_ and the system is gapless^18^. In particular, some improvements in sample quality allowed a probe of their electronic properties down to densities of ~10^8^/cm^2^, leading to the indirect observation of Coulomb interaction-induced renormalization of the Fermi velocity in graphene^19^ and the first signs of a nematic phase transition reshaping bilayer graphene’s energy band^20^.

In this work, we present the first high resolution ARPES measurements of ExBLG. We observe that neutral ExBLG is indeed gapless and shows no particular spectral features at E_D_^10^. In addition, we observe a much larger interlayer hopping energy. These observations indicate that ExBLG interacts with the substrate only weakly. In such a charge neutral BLG, a ***k***-dependent anisotropic electron-phonon (*e*-ph) coupling is observed strongly along the *K*-*Γ* direction whereas it is vanishingly small along the opposite *K*-*M* direction. More importantly, we find that the electron-electron (*e*-*e*) scattering rate scales linearly with energy over a large range of energy. This information provides, for the first time, strong evidence for non-Fermi liquid quasiparticle behavior in BLG at low energies. Although such a behavior has been theoretically studied by a number of groups^5^,^18^,^21^,^22^,^23^, there is no experimental evidence so far to support it.

Graphene samples are prepared by conventional micro-mechanical cleavage method on heavily n-doped Si substrates with native oxide only. Figure 1a to 1d show the mapping of photoemission intensity and the first derivative of the band dispersion around the *K* point taken at photon energies 54 eV and 82 eV. While measurements at 54 eV enhance the outer band and suppress the inner band, measurements at 82 eV exhibit the opposite effect^15^,^24^ (see details in Supplementary Information (SI)). The derivative plots clearly demonstrate that our ExBLG sample is free from contribution of SLG and other few-layer graphene across the entire surface area (≈50 *μm* × 30 *μm*). The cuts in momentum space (Fig. 1e) deviate from the *Γ-K*-*M* direction by 8.5° and 9.5° for 54 eV and 83 eV, respectively, and are marked as *K*-′′*Γ*′′ and *K*-′′*M*′′. Because of this small deviation, we can detect the band dispersion also from the *K-*′′*M*′′ branch, but with much lower intensity than the *K-*′′*Γ*′′ branch. The asymmetric intensity can be associated to the interference effect from the dipole matrix element^24^, which suppresses one branch of the bands. This is also reflected in the momentum distribution curves (MDCs), as shown in Fig. 1f in the vicinity of E_F_. Each curve, separated by 10 meV can be fitted by two Lorentzians with varied heights arising from the interference effect. As a guide to the eye, we connect each of the peaks by dashed lines. These two lines intersect at E_D_, which is aligned with E_F_. Such a proximity of E_D_ and E_F_ demonstrates that our ExBLG is charge neutral and free of substrate doping. Moreover, there is no gap between the π and π^*^ bands. These observations confirm a weak interaction between ExBLG and the Si substrate, which maintains the symmetry between the two layers.

To explore better the energy dispersion in ExBLG, we plot the constant energy surfaces of ARPES intensity maps in Fig. 2 with the superimposed contours calculated by the tight-binding (TB) model^25^. These constant energy contours are used to extract the TB parameters *γ*_0_, *γ*_1_, *γ*_3_ and *γ*_4,_ which are compiled in Table 1. For comparison, we list also the corresponding parameters from previous works on epitaxial BLG (EpBLG)^15^ and graphite^26^. All parameters are comparable with those reported in earlier works except the hopping integral between two layers *γ*_1_. In ExBLG (0.61 eV) it is 1.25 times that reported in EpBLG (0.48 eV)^15^. We attribute the difference to the much smaller interaction with the SiO_2_ substrate, which in turn decreases the distance between the two layers leading to an enhanced interlayer coupling^27^. Figure 1g shows the calculated density of states (DOS) based on the extracted TB parameters, which show that the DOS at E_D_ is finite, confirming the metallic behavior of BLG. In contrast, SLG and graphite have a nearly vanishing DOS at E_D_. The finite DOS in ExBLG is due to the quadratic dispersion near E_D_, but the DOS becomes linear away from E_D_. For BLG on a weakly interacting substrate, such as Si with native oxide, the quadratic band dispersion, which produces a constant DOS, is hence valid in only a small energy region around the Dirac point. This result is in agreement with earlier cyclotron resonance experiments^28^. This condition implies that ExBLG can be considered a Lorentz invariant system in the “ultra-relativistic” limit (for k > > *mv*, we have *ε*_*k*_ ≅ *vk*).

The ARPES intensity maps in Fig. 2 are modeled by a matrix element calculation based on the TB model (see SI). The basic ingredient in this calculation is the interference of matrix element components from the initial state wave functions of the four non-equivalent sites in BLG. Figure 2d shows an example of the calculated intensity map at band-energy −0.8 eV, demonstrating a good agreement with the measurement in Fig. 2b. We also calculate the band dispersion and the corresponding (matrix element) interference term squared near E_D_ (Supplementary Fig. S2). There exist three extra Dirac points very near *K* in band dispersion along the three *K*-*Γ* directions. At 82 eV the intensity of the outer π band appears strong only very near the *K* point (Supplementary Fig. S2d); moreover, very near E_D_ the intensity of the outer π* band, which becomes partially occupied due to finite temperature, appears very weak at lower *k* than *K* while strong at higher *k*. These behaviors are observed experimentally in Figs. 1c and 1f. These comparisons suggest that the experimental quasiparticle band dispersion and its transition intensity of a neutral ExBLG on a Si surface with native oxide can be described well by a single particle TB model of free-standing BLG on both high and low energy scales.

We have so far considered only the bare band dispersion; however, many-body interactions modify the single particle picture reflected in the spectral function for ARPES:


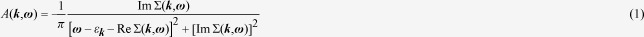


where Re Σ(***k**,ω*) and Im Σ(***k**,ω*) refers to the real and imaginary parts of the self-energy, respectively. In the presence of many-body effects, Re Σ(***k**,ω*) corresponds to the renormalization of the band energy, and 2 × Im Σ(***k**,ω*) corresponds to the scattering rate. Figure 3a shows the energy distribution curves (EDCs) near *K*, at 54 eV photon energy. The *K-*′′*Γ*′′ branch exhibits a phonon induced feature at a constant binding energy (E_B_) near 0.2 eV leading to asymmetric EDCs. A dip at this phonon energy was observed in an n-doped graphene system and interpreted as due to *e*-ph coupling^29^. It is remarkable to see the intensity of this feature relative to the main peak grow rapidly away from *K*. On the other hand, the same phonon induced feature is almost absent along *K-*′′*M*′′ and hence, the EDCs appear symmetric and Lorentzian-like. This effect strongly suggests that the *e*-ph coupling is anisotropic, robust along *K-*′′*Γ*′′ but absent along *K-*′′*M*′′. The inset shows the simulated spectral functions of an isotropic metallic system coupling to a single Einstein phonon with different coupling strength *λ*^30^,^31^. The simulation qualitatively reproduces the observed trend on the increasing weight of a phonon induced “bump” at a characteristic energy slightly larger than the phonon energy itself.

Based on the previous discussion, we can thus assume that the *e*-ph coupling is negligible along the *K-*′′*M*′′ branch. The remaining contributions for self-energy along the *K-*′′*M*′′ branch Im Σ^*K-*′′*M*′′^ are due to *e*-*e* scattering and electron-impurity (*e*-im) and defect scattering (plasmons are absent in charge neutral samples), which we can express as: 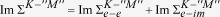. (Some contribution to *e*-im scattering might come also from rippling, which mixes *p*_σ_ and *p*_π_ bands^4^.) The same holds for the *K-*′′*Γ*′′ branch but with the addition of the *e*-ph interactions: 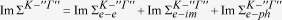. Large curvature of dispersion near E_D_, rapid variation of matrix element and the existence of phonon induced feature require us to apply both MDC and EDC to determine peak positions and widths as explained in the SI. Figure 3b shows the measured HWHM (equal to Im Σ) after deconvolution to eliminate the energy spread due to the instrumental resolution and the overall angular spread (see SI).

In our analysis, we focus on data of the outer π band with E_B_ less than 0.6 eV to avoid Auger like inter-band *e*-*e* scattering, which might complicate the interpretation. The data along the *K-*′′*M*′′ branch can be well fitted by a straight line: 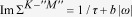, where τ is a quasiparticle lifetime that we associate with the residual disorder in the system and *b* is a constant^32^. We find that *τ* ≈ 10^−14^ s (*ħ*/2*τ* ≈ 30 meV). Since the *e*-im scattering rate is proportional to the DOS at the hole E_B_^33^,^34^ and DOS in BLG is linear in energy, the *e*-im scattering rate would also be linear in energy. Since DOS at the Dirac energy (equals Fermi energy) is finite we may scale the shape of DOS to match the finite scattering rate at E_F_ to represent the contribution from the *e*-im scattering, shown as the green line. After subtracting this part due to *e*-im scattering from the measured HWHM the resulting data displays scattering rat*e* solely from the *e*-*e* scattering. It is *e*vident that the *e*-*e* scattering rate is linear in energy, deviating completely from being quadratic as predicted by the Fermi liquid theory. This non-Fermi liquid behavior has been discussed by theoretical calculations on this honeycomb lattice^21^,^22^. In contrast, Im Σ^*K-*′′*Γ*′′^ of the opposite branch contains contributions coming from the *e*-ph interaction that produces a “bump” for band energies near E_D_ (Fig. 3a). Assuming that *e*-*e* and *e*-im interactions are the same along *K-*′′*M*′′ and *K-*′′*Γ*′′, we can extract the contribution solely due to the *e*-ph interactions: 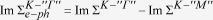, as shown in Fig. 3c. This assumption is warranted by the fact that we expect the *e*-*e* and *e*-im interactions to be short-ranged due to screening. Short range interactions produce self-energy contributions which are very weakly depend*e*nt on momentum in the Brillouin zone. The resulting *e*-ph contribution to the self-energy is comparable to that in a recent ARPES study of graphite^34^, but much smaller than an earlier study^35^. Only data with E_B_ larger than 80 meV are presented to avoid effects due to large curvature in the bare band dispersion near the Fermi level and a drastic change in matrix element (see SI).

To further prove the existence of anisotropy of the *e*-ph interactions we present in Fig. 4a the peak position determined by MDC and EDC as described in the SI overlaid with fitted TB band. It can be seen that this TB band fits data well in the *K-"M"* branch while there exists systematic deviation from peak position at low E_B_ along *K-"*Γ*"*. This TB band, being determined at high E_B_ away from the phonon influenced low E_B_ region, can serve as the bare band for extracting many-body self-energy^36^. The real part of self-energy Re ∑ due to phonons can thus be obtained as the energy difference of the measured quasiparticle band and the bare band, plotted as the data points in Fig. 4b. It can be seen that Re ∑ has a maximum around 190 meV. This shape is similar to many doped graphene systems^29^,^36^,^37^,^38^,^39^ which are metallic with a nearly linear band crossing E_F_ with a well defined k_F_, in contrast to the current neutral BLG with the near parabolic band maximum close to E_D_ (= E_F_) displayed in Fig. 4a. The absence of Re ∑ data points very near E_D_ is explained in the SI. We apply Kramers-Kronig relations to Re ∑ to obtain Im ∑ drawn as the continuous line in Fig. 4b. Thus obtained Im ∑ along *K-"Γ"* is plot in Fig. 4c with that measured directly from peak widths after subtracting out the linear widths along *K-"M"*; the agreement is quite good. This demonstrates our analysis based on the assumption of *e*-im and *e*-*e* interactions being weakly dependent on momentum catches the correct physics. Furthermore, that independently measured Re ∑ and Im ∑ satisfy Kramers-Kronig relations along *K-"Γ"* where a clear phonon induced bump appears in EDCs while phonon related self-energies are vanishingly small along *K-"M"* without trace of phonon induced bump provides strong evidence of anisotropy of *e*-ph interaction in this neutral BLG system on a Si surface with native oxide. One notes here that the bare band used for extracting phonon self-energy includes the renormalization due to *e*-*e* interaction because the linear Im ∑_*e*-*e*_ results in monotonically increasing Re ∑_*e*-*e*_ with E_B_ thus it can be absorbed in the TB fit.

It is noted that anisotropy of *e*-ph coupling strength along different directions near the *K* point has been reported in graphite^34^, with that along *KΓ* stronger than along *KM* in the same order as our data although both are finite in graphite. Anisotropy has been discussed previously in many undoped graphene and graphite related systems, their predictions are inconsistent with our data^36^,^37^,^38^,^39^,^40^,^41^,^42^,^43^ (See SI). Heavily electron-doped systems generally possess very different characteristics from undoped systems in that the former resemble closely to normal metals and Fermi-liquid like as described in the previous paragraph. On the other hand, undoped or very lightly doped systems k_F_ is either degenerate at the Dirac point or two k_F_s of small hole or electron pockets are very near one another likely making the behavior more complicated. There are reports of doped graphene exhibiting isotropic *e*-ph coupling^39^. There are also reports showing anisotropic *e*-ph coupling. For example in potassium doped graphene on Au/Ni (111)^36^ and doped graphite such as superconducting CaC_6_^44^ *e*-ph coupling is stronger along *K-M* than *K-Γ*, in opposite order to our case.

In Fermi-liquid theory the imaginary part of the self-energy scales quadratically with energy *ω* as mentioned previously. In our experiment, Im Σ_*e*-*e*_ is linear in *ω*, demonstrating neutral ExBLG should not be described with the Fermi liquid theory. Such a dependence of the self-energy with frequency is characteristic of theories involving competing orders close to quantum critical transitions^18^. Similar anomalous dependences in the self-energy are observed in 2D transition metal dichalcogenides where there is a quantum criticality associated with the competition between charge density wave (CDW) and superconductivity^45^. It is clear that BLG is much more unstable towards ordered phases than SLG, because of the enhancement in the DOS. It has been suggested by transport experiments in suspended samples that BLG has broken symmetry states with gap opening^46^. Similar experiments in supported samples^47^, however, have shown an absence of a gap but an anomaly in the quantum Hall response that can be associated with a putative nematic phase^48^,^49^.

In our experiments, with supported samples, we see direct evidence of neither a gap opening nor a phase transition. The anomalous broadening of the ARPES linewidth clearly indicates the presence of soft, low energy modes, which would be consistent with the proximity of a quantum critical point (QCP). The presence of the substrate must clearly influence, via screening, the strength of the Coulomb interactions, hence moving the system away from the critical region, in contrast with the experiments with suspended samples.

In summary, using ARPES we demonstrate that ExBLG on Si substrate with native oxide is gapless and does not behave as a Fermi liquid. We observed for the first time in such charge neutral BLG a strongly anisotropic ***k***-dependent *e*-ph coupling near *K* as well as an Im Σ_*e*-*e*_ that scales linearly with *ω* over a surprisingly large range of E_B_. This directly observed non-Fermi liquid behavior strongly supports the existence of 2D quantum critical points at the charge neutrality point, suggested by previous transport experiments showing signs of new ordered phases in BLG^20^,^46^.

## Methods

Angle-resolved photoemission measurements were conducted at the National Synchrotron Radiation Research Center in Hsinchu, Taiwan using the U9-CGM spectroscopy beamline. All samples were annealed up to 620 K for 10 minutes before measurements at 65 K. The base pressure in the chamber was 7.5 × 10^−11^ Torr. The spectra were collected by a Scienta SES-200 hemispherical analyzer with an angular resolution about 0.2° with a step of 0.125° in raw spectra for 14° angular mode. The polarization vector was always in the angular dispersive plane. The overall energy resolution was about 58 meV.

## Additional Information

**How to cite this article**: Cheng, C.-M. *et al.* Anomalous Spectral Features of a Neutral Bilayer Graphene. *Sci. Rep.* **5**, 10025; doi: 10.1038/srep10025 (2015).

## Supplementary Material

Supplementary Information

## Figures and Tables

**Figure 1:**
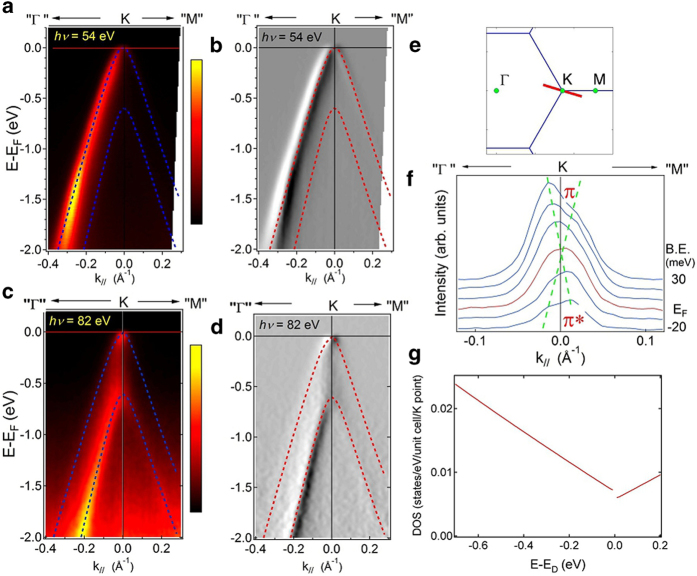
Band dispersion of bilayer graphene. **a**, False color plot of EDCs vs k_||_ at 54 eV photon energy. **b**, First derivative plot of **a**. **c**, False color plot of EDCs vs k_||_ at 82 eV photon energy. **d**, First derivative plot of **c**. The dashed lines are tight-binding fits to data. **e**, Schematic of momentum space cut of **a** and **b**; the angle to the *Γ-K-M* direction is 8.5° and 9.5° for 54 eV and 82 eV, respectively. **f**, MDCs around the Fermi energy. **g**, Calculated DOS based on the extracted tight binding parameters.

**Figure 2:**
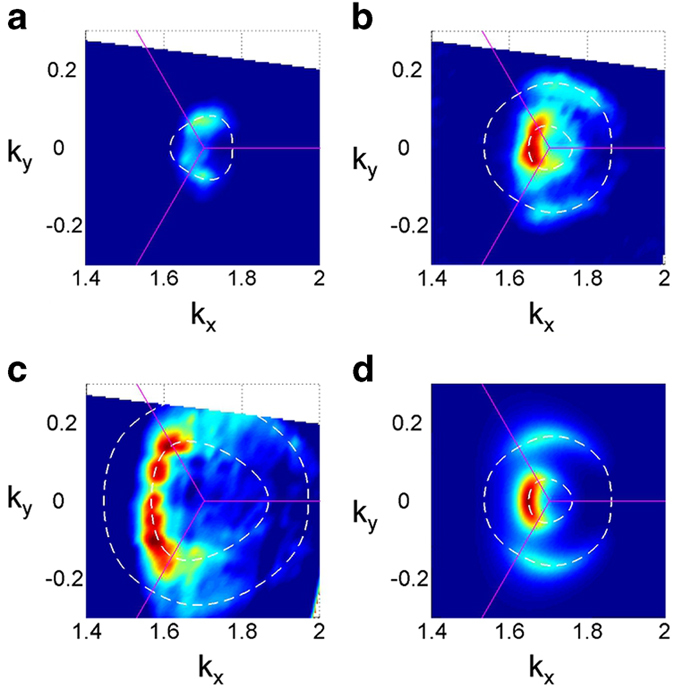
False color plots of photoemission intensity in momentum space at various constant energies at 82 eV photon energy. **a**, −0.3 eV. **b**, −0.8 eV. **c**, −1.4 eV. **d**, simulation at −0.8 eV. The dashed lines are tight-binding fits to data.

**Figure 3:**
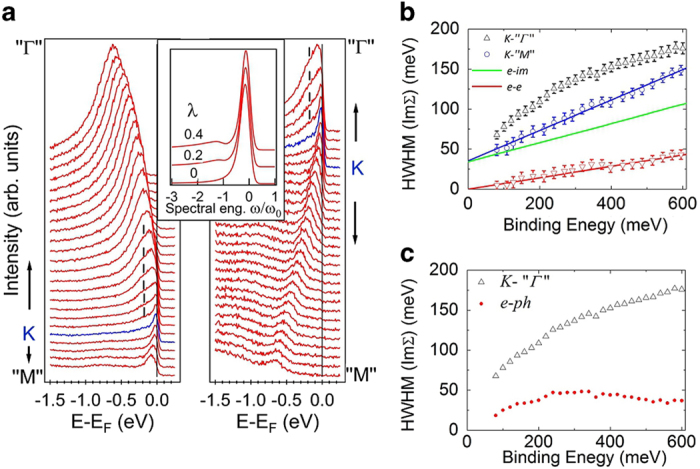
Original EDCs at 54 eV photon energy and the extracted quasiparticle widths. **a**, EDCs at 54 eV near the *K* point with each curve separated by 0.007 Å^−1^. The phonon induced bump is highlighted. The inset shows the simulated spectral functions with an energy scale normalized to an Einstein phonon ω_0_ with various coupling constants. **b**, Extracted imaginary part of self energy (HWHM) along both *K*-′′*Γ*′′ and *K*-′′*M*′′ directions. **c**, Electron-phonon interaction extracted from *K*-′′*Γ*′′ branch.

**Figure 4:**
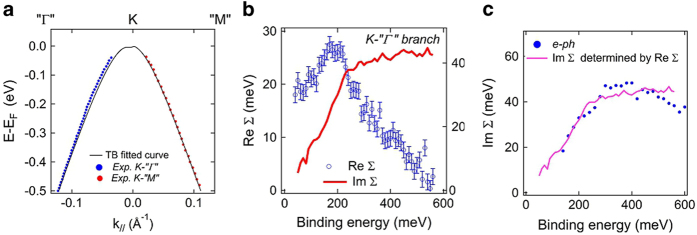
Anisotropy of self-energy. **a**, Peak positions determined by MDC and EDC are plot as dots overlaid with the TB band served as the bare band to extract self-energies. **b**, Extracted real part of self-energy Re ∑ along *K*-′′*Γ*′′ shown as open circles with error bars and the imaginary part of self-energy Im ∑ obtained by Kramers-Kronig relations from Re ∑ drawn as a continuous line. **c**. Im ∑ obtained by Kramers-Kronig relations and by measured widths responsible for the *e*-ph interactions show very good agreement.

**Table 1:** TB parameters (in eV) from the present and previous experimental works.

**TB parameters**	γ_**0**_	γ_**1**_	γ_**3**_	γ_**4**_
Pres. work	−3.21	0.61	0.39	0.15
Prev. bilayer*	−3.24	0.48		
Prev. graphite#	−3.16	0.39	0.315	0.044

^*^ARPES of BLG grown on SiC substrate, Ref. 15.

^#^Ref. 26.
