# Supplementary material for: Anomalous Spectral Features of a Neutral Bilayer Graphene
Source: Sci Rep. 2015 May 18;5:10025. doi: 10.1038/srep10025 (PMC4434949; doi:10.1038/srep10025)
Supplement: Supplementary Information [file srep10025-s1.doc]

Supplementary Information for

Anomalous Spectral Features of a Neutral Bilayer Graphene

C.-M. Cheng 1*, L. F. Xie 2,3*, A. Pachoud^2,4,5^, H. O. Moser^2,6,7^, W. Chen^2^, A. T. S. Wee^2,5^, A. H. Castro Neto 2,5, K.-D. Tsuei1,8,# & B. Özyilmaz 2,3,5,&

^1^National Synchrotron Radiation Research Center, 101 Hsin-Ann Road, Hsinchu

30076, Taiwan

^2^Department of Physics, National University of Singapore, 2 Science Drive 3,

117542, Singapore

^3^NanoCore, 4 Engineering Drive 3, National University of Singapore 117576, Singapore

^4^Graduate School for Integrative Sciences and Engineering (NGS), National University of Singapore,

28 Medical Drive, Singapore 117456

^5^Graphene Research Centre, National University of Singapore, 6 Science Drive 2,

117546, Singapore

^6^Singapore Synchrotron Light Source, National University of Singapore, 5 Research

Link 117603, Singapore

^7^Karlsruhe Institute of Technology (KIT), Network of Excellent Retired Scientists (NES) and Institute of Microstructure Technology (IMT), Postfach 3640, 76021 Karlsruhe, Germany

^8^Department of Physics, National Tsing Hua University, 101 Sec. 2, Kuang-Fu Road, Hsinchu 30013, Taiwan

1. **Sample preparation and characterization**

Graphene samples are prepared by conventional micromechanical cleavage method on (unconventional) heavily n-doped Si substrate covered with native oxide. This approach provides two main advantages. First, similar to the conventional SiO_2_/Si substrates on which almost all graphene field effect transistors are based, graphene is only weakly coupled to the substrate by van der Waals force. Hence, the effects of the graphene-substrate interactions are much reduced. Second, the presence of a thin native oxide layer insures negligible charging effects during ARPES measurements. This method thus avoids an artificial line width broadening caused by photoelectron induced charging effects on conventional (thick SiO_2_) wafers^1^ and allows us to probe the intrinsic electronic properties of nearly-free-standing graphene with only weak substrate interactions. However, due to the absence of the thicker SiO_2_ layer, we can no longer effectively utilize the conventional optical microscope to identify graphene layers as pioneered in the original experiments. What remains visible is a faint contour of few layer graphene flakes. Therefore, we first have carefully mapped such regions with optical inspection and then use Raman spectroscopy^2^ to determine the number of layers and their exact location. The optical image of graphene and its Raman spectrum is shown in Figure S1. The yellow dashed dotted line in Figure S1a encloses the area of BLG sample. The surrounding native oxide provides weak contrast for sample identification. Raman spectra (Figure S1b) were checked at the marked points to confirm BLG. Unlike epitaxial graphene whose crystal orientation is fixed to known substrate orientation and can be determined by LEED or STM prior to measurement, this is more challenging in exfoliated graphene; here an iterative approach is required. This together with the experimental set up leads to a small deviation of 8.5 degrees at 54 eV photon energy from the *Γ-K-M* direction in our measurements (marked as '′*Γ*′′*-K-*′′*M*′′), as shown in the momentum space cut (Fig. 1e). In hindsight, this accidental deviation from the *Γ-K-M* direction has been crucial to the analysis of the experimental results. It allows us to detect the much weaker band dispersion nearly along the *K*-*M* direction, essential for isolating different contributions to many-body effects from the overall scattering rate. We note that the measured narrow MDC width 0.039 Å^-1^ in our BLG is comparable to that measured at E_F_ of SLG appeared in the literature suggesting high quality of our data^3-5^.

1. **Analytical band dispersion of bilayer graphene**

Following the tight binding (TB) description^6^ of a free-standing BLG while keeping only the in-plane nearest neighbor hopping integral *γ*_0_, out-of-plane nearest neighbor hopping integral *γ*_1_ and next nearest neighbor up to *γ*_3_, and assuming the on-site potential of the two layers being +*V*/2, one obtains an analytical form of band dispersion for bilayer graphene

$$E^{2}=\frac{\gamma_{1}^{2}}{2}+\frac{V^{2}}{4}+(\gamma_{0}^{2}+\frac{\gamma_{3}^{2}}{2})|f|^{2}\pm\sqrt{\Gamma}$$

$$\Gamma=\frac{1}{4}(\gamma_{1}^{2}-\gamma_{3}^{2}|f|^{2})^{2}+\gamma_{0}^{2}|f|^{2}(\gamma_{1}^{2}+V^{2}+\gamma_{3}^{2}|f|^{2})+\gamma_{0}^{2}\gamma_{1}\gamma_{3}(f^{3}+f*^{3})$$

where *f* = Σ*_j_* exp(*i****k*.*δ****_j_*), ***δ****_j_* are vectors to the nearest neighbor atoms. Expanding ***k*** near the *K* point ***K*** = (2π/*a*)(2/3, 0), and the *K*′ point –***K***, ***k*** =±***K***+Δ***k***, $f=\mp\frac{\sqrt{3}}{2}a|\Delta k|\exp(\mp i\theta_{\Delta k})$, *θ*_Δ_***_k_***= tan^-1^(Δ*k_y_*/Δ*k_x_*), one obtains the formula by McCann and Fal’ko^7^. A finite on-site potential difference *V* opens up a gap of a similar magnitude around the Dirac energy *E_D_*=0 and a neutral system is an insulator. In our case of nearly-free-standing neutral BLG *V* is set to zero thus a vanishing gap. One can obtain with a further approximation an expression for the two low lying bands ^8^.

$$E^{2}=|f|^{2}\left[\gamma_{3}^{2}\pm2\frac{\gamma_{0}^{2}\gamma_{3}}{\gamma_{1}}|f|\cos(3\theta_{\Delta k})+\frac{\gamma_{0}^{4}}{\gamma_{1}^{2}}|f|^{2}\right]$$

It can be seen that in addition to the usual Dirac point at *K* (*K*′), where *E*=0 and | *f* |=0, there exist three extra Dirac points at cos(3*θ*_Δ_*_k_*)=1 and | *f* |≠0 (*γ*_1_*γ*_3_>0), along the three *K*-*Γ* directions. The influence of the parameter *γ*_4_ on the low-energy bands is much smaller than it of *γ*_3_, and *γ*_4_ only lifts the electron-hole symmetry slightly. The existence of these extra Dirac points is revealed in intensities of ARPES measurements as described in the next section. It is noted here that *γ*_4_ is generally smaller than *γ*_3_, and the influence of the parameter *γ*_4_ on the low-energy bands is much smaller^9^. All numerical calculations based on TB parameters in this paper include *γ*_4_.

1. **Width analysis by momentum distribution curves and energy distribution curves in the presence of nonlinear dispersion or matrix element effect**

We discuss in this section the difficulty on extracting many body self-energies on analyzing our data by usually adopted momentum distribution curves (MDCs) compared to energy distribution curves (EDCs). In performing MDC or EDC analysis the experimental spectra are presented as an intensity map of E_B_ and a cut in momentum space ***k***=***k***_||_, $I(k,E_{B})$, as in Fig. 1. The intensity of ARPES spectra is governed by the product of matrix element squared and spectral function^10^, $I(k,E_{kin})\propto|M_{f,i}^{k}|^{2}A(k,\omega)$. In many cases the matrix elements are taken as a constant and omitted in the subsequent analysis then the ARPES intensity map becomes the intensity map of spectral functions. The spectral function *A*(***k***,ω) in the form of Eqn. (1) is by default expressed as an EDC with a fixed ***k*** and its peak position in *ω* as a function of ***k*** is denoted as the dispersion relation *E*=*ω*(***k***). MDC on the other hand is extracted at constant *ω* ^3^ in the intensity map of spectral functions.

Presume one can measure ARPES spectral functions with infinite energy and angular resolution. Each EDC is supposed to have a Lorentzian lineshape with its HWHM representing the imaginary part of self-energy ImΣ. An MDC is essentially taking different parts of various spectral functions (EDCs) with different k and composing them together. The width in MDC is then converted to the width in EDC, which is of prime interest, by multiplying the dispersion slope ΔE/Δk. If the quasiparticle band dispersion is linear and the change of Im Σ is small within the energy range of the width (or the k-independence of self-energy Σ(***k***,*ω*) ≈ Σ(*ω*) ^3^) the MDC will also appear perfectly Lorentzian. MDC analysis bears advantages over EDC in the sense that the former is not influenced by Fermi function cutoff and secondary electron tail at lower kinetic energies. Thus MDC analysis is widely used in extracting self-energy from high resolution ARPES spectra.

However, MDC bears an intrinsic problem when the quasiparticle band dispersion is not linear. In that case the MDC will appear to be asymmetric with a larger HWHM on the side where the peak disperses closer to the original energy of interest. This becomes a very severe problem to determine the width by MDC when the dispersion deviates very much from being linear, for example near the extremum of a parabola such as the band dispersion of BLG near the Dirac energy (equals the Fermi energy in neutral BLG). Under this condition EDC becomes advantageous over MDC as the former stays Lorentzian.

The appearance of a phonon induced bump at the lower kinetic energy side when a peak disperses to have a binding energy *E_B_* smaller than the phonon characteristic energy 200 meV in the *K-"Γ"* branch posts a problem to utilize EDC to determine its width. MDC must be utilized.

However, the assumption of constant matrix elements breaks down in general in graphene related systems^11,12^ as will be elaborated. When the matrix element effect is prominent, as in our case near the Dirac energy and taking data along a momentum space cut near the *Γ-K-M* line in graphene related systems, an MDC becomes distorted with a smaller HWHM on the side when matrix element squared becomes quickly diminishing. An EDC on the other hand, has an advantage over an MDC as the former stays Lorentzian.

Nonlinear band dispersion, non-negligible matrix element effect and the appearance of phonon induced bump in spectral functions contribute to limiting our capability to reliably determine the width. The peak position in contrast is much easier to locate and can be checked by both MDC and EDC. Width determination is usually not a problem in doped graphene which is metallic with a clear mark of Fermi momentum where the band disperses crossing the Fermi energy and the dispersion is very linear^13,14,15^.

In summary widths extracted by MDC and EDC are consistent from peaks with *E_B_* larger than 200 meV; below 200 meV MDC must be used in the *K-"Γ"* branch due to the presence of phonon induced bump at 200 meV; both MDC and EDC may be used to extract widths in the *K-"M"* branch with consistent results until the band dispersion curvature becomes too large where band energy too near the Dirac energy (Fermi energy).

We now discuss the origin of matrix element effect. Neglecting Umklapp process, ***k****_f_* _||_ = ***k***, the matrix element for BLG can be expressed as , where ***k****_f_* is the momentum of final state inside solid, ***ε *** the polarization unit vector, *ϕ*(***r***) the atomic wave function, and the interference term from four sites $\tilde{M}_{f,i}^{k}=[a_{1}(k)+b_{1}(k)]+[a_{2}(k)+b_{2}(k)]e^{\pm i k_{f z} d}$, where *a*_1_, *b*_1_ and *a*_2_, *b*_2_ are the (complex) coefficients of Bloch states of A and B sites of layers 1 and 2, respectively, of TB wave functions at ***k***, *d* is the interlayer spacing; the + sign refers to different sequences of stacking and an average is calculated in the following simulations. Figure 2d presents the simulated $|\tilde{M}_{f,i}^{k}|^{2}$ for -0.8 eV band energy as an example, assuming an inner potential of 17 eV^16,17^ and a *d* spacing of bulk graphite 3.354 Å. The agreement with experimental data in Fig. 2b is rather satisfactory. The interference term squared varies dramatically along the momentum space cut in experiment as demonstrated in Fig. S2. The calculated TB band energy dispersion along the *Γ*-*K*-*M* line very near the *K* point is shown in Fig. S2a; there exist three extra Dirac points along three *K* to *Γ* directions as shown in the previous section. A gap opens up slightly away from this direction as presented in Fig. S2b for an angle 8.5^o^. The calculated interference term squared mimicking experimental conditions of 8.5^o^ at 54 eV and 9.5^o^ at 82 eV photon energies are shown in Figs. S2c and S2d, respectively. It can be seen in Fig. S2d that at 82 eV the intensity of the inner π band decreases rapidly across the *K* point from smaller momentum while the outer π band appears strong in intensity only very near the *K* point, which is due to the nearby extra Dirac point; both are observed experimentally in Figure 1c. At 54 eV in Figure S3c the intensity of the inner π band almost completely vanishes while the outer π band appears strong in intensity at momentum less than the *K* point while quickly diminishes at larger momentum. At experimental finite temperature the outer π* band becomes partially occupied and its intensity alternates with that of the outer π band. These behaviors are observed in Figs. 1a, 1f and 2a. These observations suggest the experimental quasiparticle band dispersion and its transition intensity of an ExBLG on a Si surface with native oxide can be described well by a single particle TB model of free-standing neutral BLG on both high and low energy scales, and further suggest the ExBLG is neutral without gap opening within our experimental resolution.

1. **Deconvolution of contributions to energy width from finite energy and angular resolution and sample rippling**

A finite angular spread contributes to the energy width by band dispersion |Δ*E*|=|d*E*/d*k*|Δ*k*, where , in addition to a finite instrumental energy resolution. This part of energy width is the largest away from the *K* point where the dispersion is large while vanishing near *K* where the dispersion vanishes. The total angular spread Δ*θ* is a convolution of instrumental angular resolution, which is about 0.2°, and rippling of sample surface. The sample rippling was not known *a priori* but can be determined in the following process. We find the Gaussian deconvoluted Lorentzian width is always linear in energy for all reasonable assumption of sample rippling. The EDC nearest to *K* can be normalized to a function convoluted with a Fermi function of known temperature and a Gaussian representing measured energy resolution to yield an experimental spectral function without the influence of Fermi function. This thus obtained peak width is a convolution of finite energy resolution and quasiparticle self energy width. The overall angular spread Δ*θ* is chosen to match the intercept at the Fermi energy of a linear fit to the Lorentzian width at larger binding energies to the deconvoluted quasiparticle self-energy width. The obtained total angular spread 0.46° is primarily from sample rippling. This sample rippling causes a large *k*_||_ smearing in EDCs shown in Fig. 3a. The strong ***k***-dependence of phonon induced feature in EDCs would be more dramatic had the rippling removed. For example, there would be no phonon induced feature in EDC at the *K* point. The largest uncertainty in this analysis is the estimation of peak width in experimental spectral function in the vicinity of *E_F_*. This analysis assures that the quasiparticle scattering rate combining electron-electron (*e*-*e*) scattering and electron-impurity (*e*-*im*) or defect scattering is linear in energy. This is one of the most important conclusions in this study. We also note that sample surface rippling can increase the measured MDC width significantly at *K* (Fig. 1f) because the dispersion is flat.

1. **Anisotropy of electron-phonon coupling in *k*-space**

We discuss in this section a possible origin of the observed strong ***k***-dependent anisotropy in the phonon induced feature. A general form of electron self energy from *e*-ph coupling can be expressed as^18^

where *g* is the *e*-ph coupling matrix element, *ε****_k_***' is a bare electron energy with a momentum ***k***', *ω****_q_***_λ_ is phonon energy with a momentum ***q***=+(***k***'-***k***) and branch *λ*, *f* and *n* are Fermi-Dirac function and Bose-Einstein function, respectively. The *e*-ph coupling matrix element *g* can be expressed as

where *i*, *j* are band indices, Δ*V****_q_****_λ_* is the *e*-ph scattering potential, and total momentum must be conserved ***q***=+(***k***'-***k***). Moreover, the energy denominator in self energy does not require total energy being strictly conserved in the scattering process^18^. Thus even for a photohole with an energy *ω*_0_<*ε****_k_***<0 not enough to excite an Einstein phonon *ω*_0_, virtual excitations can still contribute to the self energy generating a phonon induced bump in spectral function. This is a many body effect. Thus the anisotropy of *g* dictates the observed strong ***k***-dependent anisotropy in EDCs. Theoretically anisotropy of *g* in ***k***-space was predicted in graphite for intra-valley process involving phonons near *Γ *^19^ with nodes appearing a period of π, in contradiction with our observation of anti-node and node along the *Γ*-*K*-*M* line. An angular dependence of *g* for inter-valley process coupling to a phonon near *K* was also predicted in graphene and graphite^20^ but the node and anti-node directions are opposite to our observation. A recent first-principles calculation on doped and undoped graphene concludes that the angular dependence of the self-energy is insignificant at fixed energy, and the *e*-ph coupling parameter λ is isotropic in *k*-space^21^, also inconsistent with our observation. This calculation suggests the dominant phonon mode in e-ph coupling is the 200 meV one (LO) near *Γ*^21^. A more recent first-principles calculation on *e*-ph interactions in neutral free-standing few-layer (including bilayer) graphene finds that the high-frequency optical-phonon modes couple with different valence and conduction bands, giving rise to different *e*-ph interaction strengths for these modes; the magnitude squared |*g*|^2^ from some electronic band transitions involving optical modes at *Γ* are smoothly varying over a large range of electronic momentum *k* around *K* while others becomes vanishingly small near *K*, but none shows strong anisotropy^22^. That the observed phonon induced feature appears very near the phonon energy at *Γ* suggests the TO phonon mode at *K*, which has a smaller energy, may also participate in the coupling in the inter-valley scattering process. The discrepancy of *e*-ph coupling of our experiment (anisotropic) with theoretical calculations is in sharp contrast to the good agreement between the measured band dispersions and intensity maps, and the corresponding values calculated from TB models of free-standing BLG. Further investigations are necessary to clarify the discrepancy.

1. **Phase space of photohole decay by electron-hole pair creation**

The band dispersion of BLG approaches parabolic very near the Dirac energy (*E_D_* = *E_F_*) thus the phase space of photohole decay by creation of electron-hole (*e*-*h*) pair no longer vanishes, unlike SLG and graphite which have a nearly linear dispersion thus a nearly vanishing phase space^23^. A photohole is created with a momentum ***k***_0_ and an energy *ω*_0_<0 as shown in Fig. S3a. It may be filled by another electron with a higher energy *ω*_0_'<0 and a momentum ***k***_0_'. The energy difference Δ*ω* = *ω*_0_'-*ω*_0_ >0 is transferred to excite an electron with an energy *ω_h_*<0 across E_D_ to an empty state *ω_e_*>0 creating an *e*-*h* pair. The whole allowed transitions with various combinations of *ω_h_* and *ω_e_* for a given excitation energy Δ*ω* is shown in the shaded disk in Fig. S3b as a continuum in the Δ*ω* vs. Δ***k*** plot. In addition the momentum transfer Δ***k*** = ***k***_0_'-***k***_0_ must be conserved. Therefore, the phase space for the decay of a photohole at ***k***_0_ with an excitation energy Δ*ω* is the intersection shown as an arc. The total phase space is thus the integration over all possible Δ*ω*. If both the valence and conduction bands are parabolic with the same magnitude of effective mass the phase space can be calculated analytically to be proportional to |*ω*|^1.5^. For BLG the band dispersion is not isotropic in ***k*** (relative to ***K***) and the total phase space depends upon the direction. The numerically calculated *e*-*h* pair creation continuum boundary (filled above) is presented in Fig. S4a. It contains three-fold symmetry for a given excitation energy Δ*ω*, and the local minimal momentum transfer |Δ***k***| occur at 0° and 60°, and approximate maxima at 28° and 92°, with respect to the *Γ*-*K*-*M* direction. The numerically calculated phase space is presented in Fig. S4b with various direction angles relative to the *Γ*-*K*-*M* direction. The values along 9.5° (*K*-'′*M*′′) and 189.5° (*K*-'′*Γ*′′) are very close to those along 0° (*K*-*M*) and 180° (*K*-*Γ*), respectively, and the difference is negligible. The calculated phase space can be fit well with c|*ω*|*^α^*, with the exponent *α*=1.29 and 1.38 for along *K*-*M* and *K*-*Γ*, respectively, and the relative ratio of the scaling factors c of the latter to the former being 0.92. The difference is small such that we can assume Im Σ*_e-e_* are similar along *K-*'′*M*′′ and *K-*'′*Γ*′′) in the main text. One notes here that the usual criterion of abiding by Fermi liquid theory is having Im Σ being proportional to energy *ω*^2^, which is derived from pure phase space consideration of *e*-*h* pair creation in *e*-*e* scattering on an isotropic 3D free electron system, assuming a constant scattering matrix element. In 2D it is modified as proportional to *ω*^2^|ln(*ω*)| ^24^. That the calculated phase space of *e*-*e* scattering due to *e*-*h* pair creation in band structure scales faster than linear in energy in contrast to experimental observation of being linear demonstrates the importance of Coulomb interaction in scattering matrix elements, further highlights the non-Fermi liquid behavior.

**Supplemental References**

1. Knox, K. R. et al. Spectromicroscopy of single and multilayer graphene supported by a weakly interacting substrate. *Phys. Rev. B* **78,** 201408(R) (2008).
2. Wang, Y. Y. et al. Raman studies of monolayer graphene: The substrate effect. *J. Phys. Chem. C* **112,** 10637-10640 (2008).
3. Bostwick, A. et al. Quasiparticle dynamics in graphene. *Nature Phys.* **3,** 36 (2007).
4. Zhou, S. Y. et al. Substrate-induced bandgap opening in epitaxial graphene. *Nature Mater.* **6,** 770-775 (2007).
5. Rotenberg, E. et al. Origin of the energy bandgap in epitaxial graphene. *Nature Mater.* **7,** 258-260 (2008).
6. Grüneis, A. et al. Tight-binding description of the quasiparticle dispersion of graphite and few-layer graphene. *Phys. Rev*. *B* **78,** 205425 (2008).
7. McCann, E. & Fal’ko, V. Landau-level degeneracy and quantum hall effect in a graphite bilayer. *Phys. Rev. Lett*. **96,** 086805 (2006).
8. Koshino, M. & Ando T. *Phys. Rev.* *B* **73,** 245403 (2006).
9. Castro Neto, A. H., Guinea, F., Peres, N. M. R., Novoselov, K. S. & Geim, A. K. The electronic properties of graphene. *Rev. Mod. Phys*. **81,** 109-162 (2009).
10. Damascelli, A., Hussain, Z., Shen, Z.-X. Angle-resolved photoemission studies of the cuprate superconductors, *Rev. Mod. Phys.* **75,** 473-541 (2003).
11. Shirley, E. L.et al. Brillouin-zone-selection effect in graphite photoelectron angular distributions. *Phys. Rev. B* **51,** 13614-13622 (1995).
12. Mucha-Kruczyński, M. et al. Characterization of graphene through anisotropy of constant-energy maps in angle-resolved photoemission. *Phys. Rev. B* **77,** 195403 (2008).
13. Fedorov, A.V. et al. Observation of a universal donor-dependent vibrational mode in graphene. *Nature Comm.* **5,** 3257 (2014).
14. Haberer, D., et al. Anisotropic Eliashberg function and electron-phonon coupling in doped graphene. *Phys. Rev.* B **88,** 081401(R) (2013).
15. Grüneis, A., et al. Electronic structure and electron-phonon coupling of doped graphene layers in KC_8_. *Phys. Rev.* B **79,** 205106 (2009).
16. Ohta, T. et al. Interlayer interaction and electronic screening in multilayer graphene investigated with angle-resolved photoemission spectroscopy. *Phys. Rev. Lett.* **98,** 206802 (2007).
17. Zhou, S.Y., Gweon, G. H., & Lanzara, A. Low energy excitations in graphite: The role of dimensionality and lattice defects, *Ann. Phys.* **321,** 1730-1746 (2006).
18. Grimvall, G. *The Electron-Phonon Interaction in Metals*. (North-Holland, Amsterdam: 1981)
19. Jiang, J. et al. Electron-phonon interaction and relaxation time in graphite. *Chem. Phys. Lett.* **392,** 383 (2004).
20. Piscanec, S. et al. Kohn anomalies and electron-phonon interactions in graphite. *Phys. Rev. Lett.* **93,** 185503 (2004).
21. Park, C.-H. et al. Velocity renormalization and carrier lifetime in graphene from the electron-phonon interaction. *Phys. Rev. Lett.* **99**, 086804 (2007).
22. Yan, J.A., Ruan, W. Y. & Chou, M. Y. Electron-phonon interactions for optical-phonon modes in few-layer graphene: First-principles calculations. *Phys. Rev. B* **79**, 115443 (2009).
23. Leem, C. S. et al. Effect of linear density of states on the quasiparticle dynamics and small electron-phonon coupling in graphite. *Phys. Rev. Lett*. **100**, 016802 (2008).
24. Hodges, C., Smith, H., & Wilkins, J. W. Effect of Fermi Surface Geometry on Electron-Electron Scattering. *Phys. Rev. B* **4**, 302 (1971).


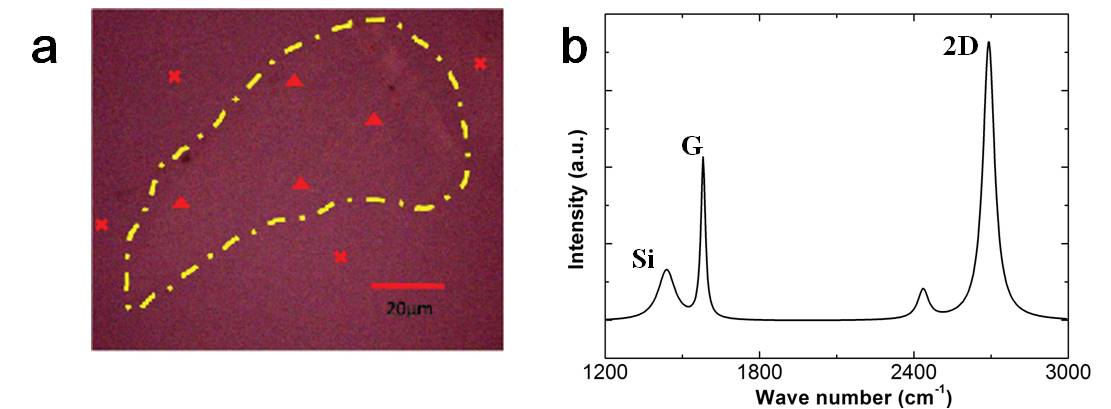


**FIG. S1. Optical image and Raman spectrum of sample. (**a) Optical image of the sample region characterized by ARPES and Raman. Yellow dashed dotted line surrounds the area, which under optical inspection indicated the presence of few layer graphene. The native oxide does give much reduced contrast for layer identification. Markings on the picture indicate the positions at which Raman imaging has been performed: red triangles indicate the region where bilayer signal is observed; red crosses indicate the locations where a Si signal is observed. (b) Raman spectrum of measured sample. The relative height of the 2D and G peaks shows the BLG property.


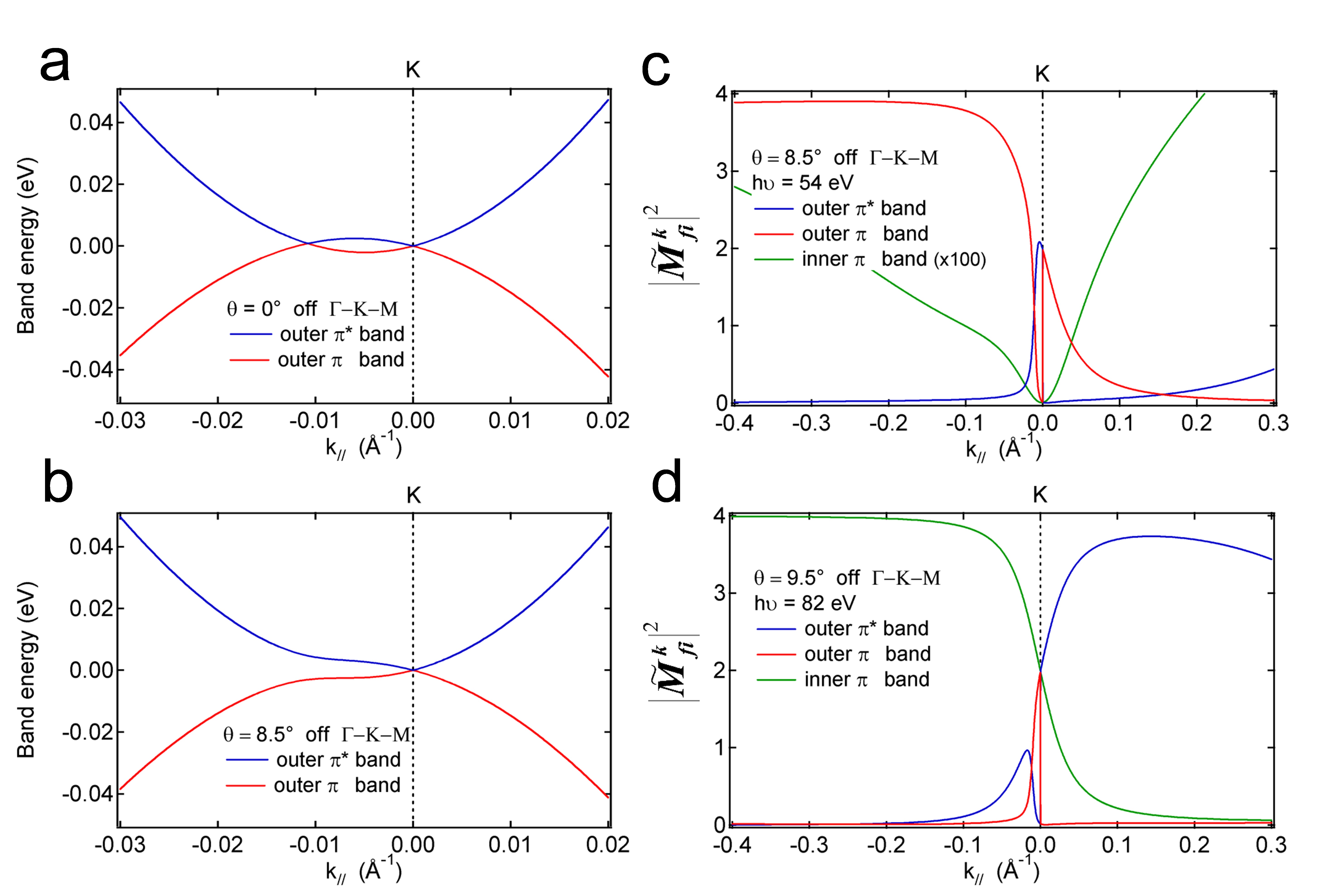


**Figure S2 | Simulated band dispersion and interference term squared of photoemission matrix elements in experimental conditions. a**, Band dispersion very near the *K* point along the *Γ*-*K*-*M* direction. **b**, 8.5^o^ off the *Γ*-*K*-*M* direction. **c**, Simulated interference term squared at 8.5^o^ off *Γ*-*K*-*M* at 54 eV photon energy. **d**, At 9.5^o^ off *Γ*-*K*-*M* at 82 eV photon energy.


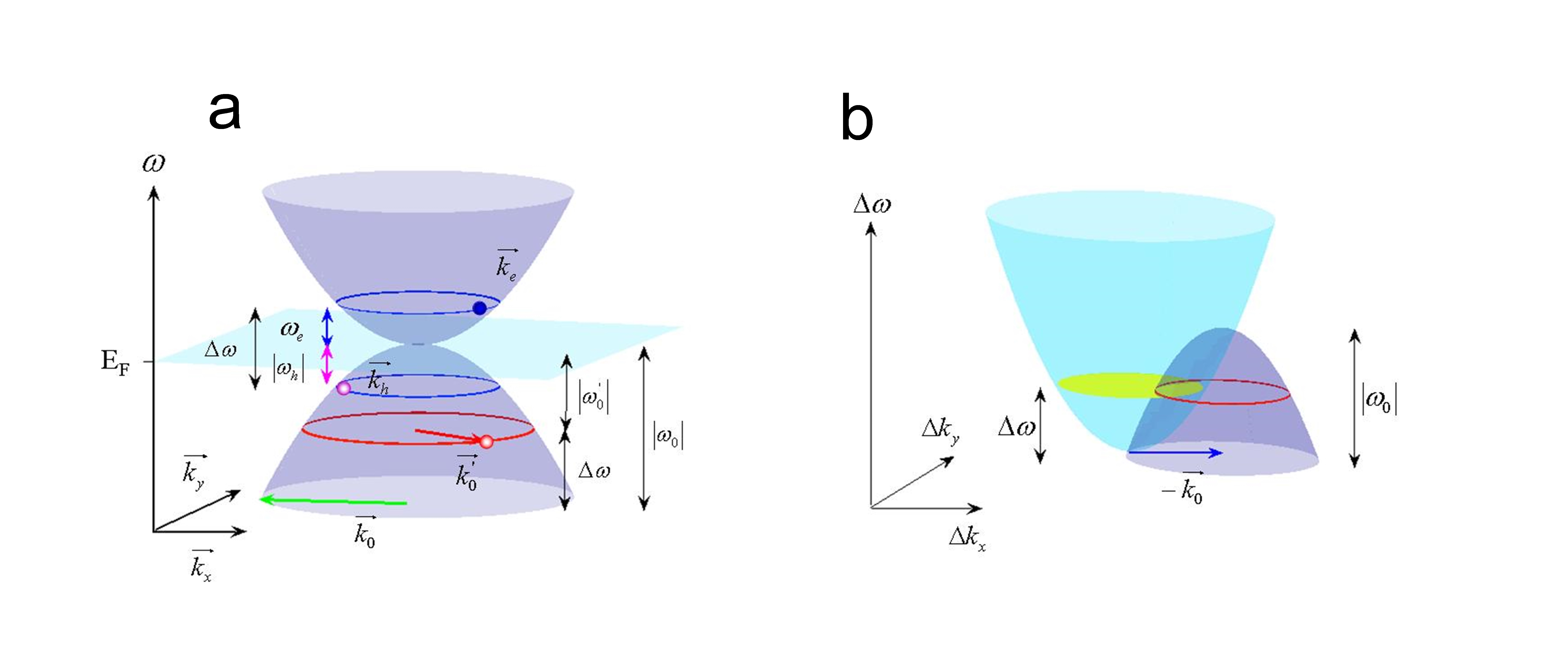


**Figure S3 | Schematic of photohole decay via electron-hole pair creation and the corresponding phase space in bilayer graphene. a**, Schematic of electron-hole pair creation (*k_h_ -> k_e_*) and transition of a photohole (*k*_0_ -> *k*_0_') in bilayer graphene. *ω_e_* and |*ω_h_*| represents the electron and hole energies, respectively. **b**, Electron-hole pair creation continuum (filled yellow disk) at an excitation energy Δ*ω* and photohole transition (open red circle). Their intersection (an arc) indicates the phase space of allowed transitions at Δ*ω*.


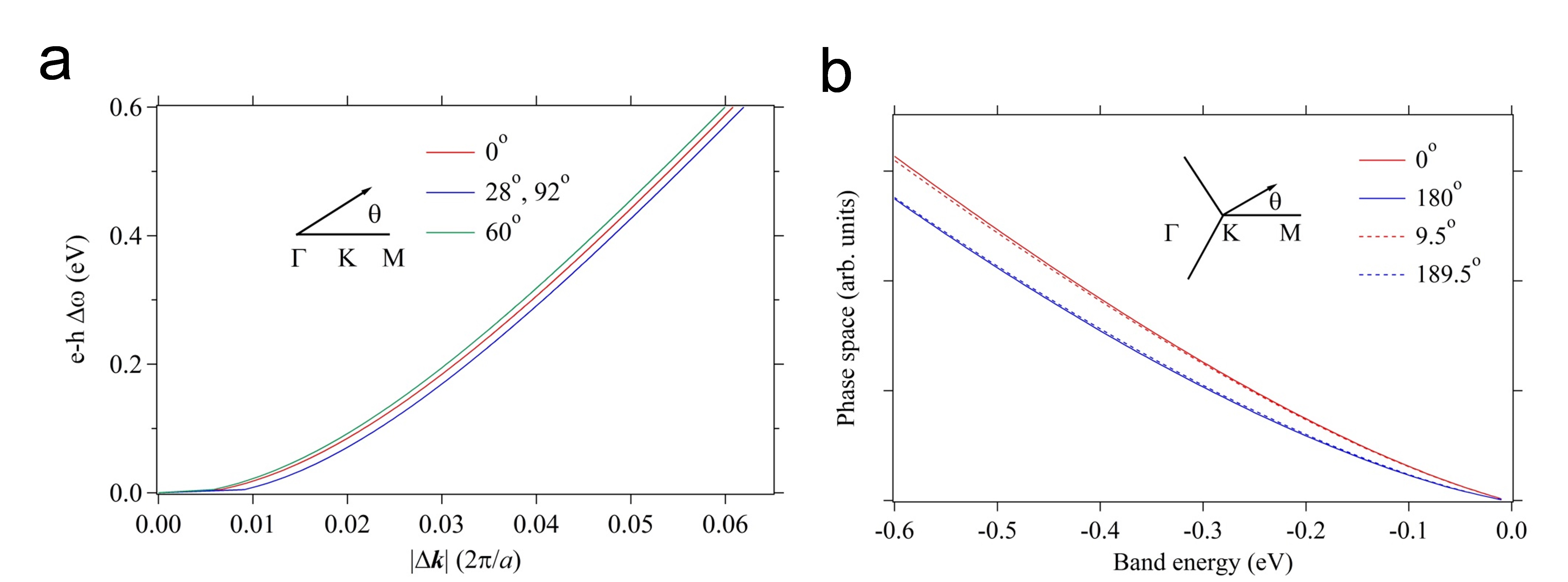


**Figure S4 | Calculated electron-hole pair creation continuum and phase space of photohole decay in bilayer graphene. a**, Calculated electron-hole pair creation continuum boundary (filled above) for a given excitation energy Δ*ω* along the local minimal momentum transfer |Δ***k***| for 0° and 60°, and approximate maxima for 28° and 92°, respectively, with respect to the *Γ*-*K*-*M* direction. It contains three-fold symmetry. **b**, Calculated phase space of electron-hole pair creation in neutral bilayer graphene along various directions of momentum ***k*** (relative to ***K*** and the *Γ*-*K*-*M* direction). 0° and 180° denote along *K*-*M* and *K*-*Γ*, respectively.
